# First Report of Generalized Face Processing Difficulties in Möbius Sequence

**DOI:** 10.1371/journal.pone.0062656

**Published:** 2013-04-24

**Authors:** Sarah Bate, Sarah Jayne Cook, Joseph Mole, Jonathan Cole

**Affiliations:** 1 Psychology Research Centre, Bournemouth University, Poole, United Kingdom; 2 Dorset Healthcare, University Foundation Trust, Bournemouth, United Kingdom; 3 Department of Neuropsychology, Wessex Neurological Centre, Southampton General Hospital, Southampton, United Kingdom; Royal Holloway, University of London, United Kingdom

## Abstract

Reverse simulation models of facial expression recognition suggest that we recognize the emotions of others by running implicit motor programmes responsible for the production of that expression. Previous work has tested this theory by examining facial expression recognition in participants with Möbius sequence, a condition characterized by congenital bilateral facial paralysis. However, a mixed pattern of findings has emerged, and it has not yet been tested whether these individuals can imagine facial expressions, a process also hypothesized to be underpinned by proprioceptive feedback from the face. We investigated this issue by examining expression recognition and imagery in six participants with Möbius sequence, and also carried out tests assessing facial identity and object recognition, as well as basic visual processing. While five of the six participants presented with expression recognition impairments, only one was impaired at the imagery of facial expressions. Further, five participants presented with other difficulties in the recognition of facial identity or objects, or in lower-level visual processing. We discuss the implications of our findings for the reverse simulation model, and suggest that facial identity recognition impairments may be more severe in the condition than has previously been noted.

## Introduction

Möbius sequence is a rare congenital condition characterized by complete (or near complete) bilateral facial paralysis and impaired bilateral movement of the eyes [1], [2]. It is not progressive and occasionally the deficits in paralysis can partially improve. The syndrome is associated with underdevelopment of the sixth and seventh cranial nerve nuclei, which occurs early in prenatal life [1]. While the sixth cranial nerve controls the abduction of the eyes, the seventh cranial nerve controls the muscles used to generate facial expressions, eye closure and lip speech. Hence, people with Möbius sequence are unable to produce facial signals, and have an immobile face characteristic of the condition. They also do not usually move their eyes in their heads, due to the sixth nerve palsy and frequent additional gaze palsies. Other common problems include cranial nerve deficits affecting the tongue and swallowing, poor coordination (due to long tract problems in the brain stem) and limb abnormalities, club feet, and missing or underdeveloped fingers or hands [3], [4].

In recent years some researchers have investigated the extent to which individuals with Möbius sequence can recognize facial expressions of emotion. Some of these studies were carried out in response to evidence for the existence of ‘mirror neurons’, where the same brain regions appear to be activated during action generation and the observation of others’ actions (e.g. [5], [6], [7]). It has been suggested that mirror neurons are involved in emotional expression recognition and empathy [8], [9]. For instance, embodied simulation accounts of facial expression recognition suggest that perceivers recognize the emotions of others by implicitly simulating the emotional experience within themselves [10]. This might occur via the perceiver unconsciously mimicking the observed expression and receiving proprioceptive feedback from the facial muscles, or by the implicit initiation of the motor programme that produces a particular expression [10], [11], [12], [13], [14]. Indeed, evidence suggests that perceivers spontaneously and covertly initiate implicit motor programmes when viewing emotive faces [15], [16], [17], [18], and the ‘facial feedback hypothesis’ posits that proprioceptive feedback from facial expressions is either necessary or sufficient for the understanding of another person’s emotional state [19], [20], [21]. Because individuals with Möbius sequence have facial paralysis and hence cannot mimic facial expressions (and presumably cannot initiate the motor programmes responsible for the production of emotional expressions), investigation of their ability to recognize facial expressions is an innovative test of embodied simulation accounts of expression recognition.

Three investigations to date have examined facial expression recognition in Möbius sequence. In an early study, Giannini et al. [22] examined facial expression processing in one individual with Möbius sequence who was of above-average intelligence and had no perceptual impairments. The authors showed the participant a series of videotapes, each displaying someone playing a slot machine for different ‘jackpot’ prizes. The participant was required to use changes in the players’ facial expression to estimate which of three jackpots the person was playing for. Whereas 300 control participants completed the task successfully, the Möbius participant was completely unable to perform the task. Moreover, she informed the experimenters that she could not interpret facial expressions in everyday life. These findings suggest that the production of facial expressions may indeed be linked to their perception.

More recently, Bogart and Matsumoto [23] carried out an on-line assessment of facial expression recognition in a larger sample of Möbius participants. The authors tested 37 adults with Möbius sequence in an Internet-based study where participants completed a facial expression recognition test. Specifically, participants were asked to label the emotional expression depicted on each of 42 faces, selecting their responses from the options ‘anger’, ‘contempt’, ‘disgust’, ‘fear’, ‘happiness’, ‘sadness’, ‘surprise’, ‘neutral’ or ‘other’. Participants were also asked to complete a Facial Expression Communication Questionnaire, which assessed their ability to communicate each of the seven facial expressions used in the recognition test. The authors found that the Möbius individuals did not differ from the control group or normative data in their emotion recognition accuracy, and additionally that accuracy was not related to the extent of their self-reported ability to produce facial expressions. Thus, these results do not support the hypothesis that reverse simulation with facial mimicry is necessary for facial expression recognition.

A more in-depth assessment of facial processing abilities was reported by Calder et al. [24], who investigated three individuals with Möbius sequence. First, the authors examined the ability to recognize facial expressions. In a basic emotional expression labelling task, none of the three participants was impaired. However, in a more demanding task that required the recognition of ambiguous emotional expressions, one participant was impaired, one was borderline impaired, and the other was unimpaired. Contrary to the most extreme interpretation of the reverse simulation model (which would suggest that facial mimicry is necessary for facial expression recognition), Calder and colleagues provided evidence that people with Möbius sequence are able to recognize basic facial expressions, despite some difficulties in a more complex task. The authors suggest an alternative interpretation of their findings, in that the impairments observed in processing facial expression may in fact be secondary consequences of Möbius sequence, resulting from eye movement abnormalities that create generalized problems in looking at faces. Indeed, the nature of Möbius sequence means that sufferers will often have less expertise and experience with faces than neurologically intact individuals of a comparable age.

While a mixed pattern of findings has been reported regarding the expression recognition capabilities of individuals with Möbius sequence and their implications for reverse simulation accounts, no work to date has explored whether these individuals can *imagine* facial expressions. Theoretically this an important issue, as some authors suggest that motor responses also play an important role in emotional facial imagery [25], [26], [27], and previous work has reported a correlation between emotional face tasks of perception, expression and imagery [28]. According to this viewpoint, an impairment in the required motor apparatus necessarily results in defective imagery as well as defective perception of facial expressions, yet this issue has not yet been explored in individuals with Möbius syndrome despite its theoretical importance. Indeed, if corresponding deficits were found in tasks assessing the perception and imagery of facial expressions, this would not only provide support for reverse simulation accounts of expression processing, but would also support the proposed link between expression perception and expression imagery.

In the current study, we carried out a further test of the embodied simulation theories by examining the ability of six Möbius participants to recognize and imagine facial expressions. This is a pertinent issue as an inconsistent pattern of findings has emerged in the three expression recognition studies reported to date, and expression imagery has not yet been examined in the condition. In addition, we investigated whether any deficits may be attributed to more generalized perceptual difficulties (i.e. those resulting from the absence of eye movements), rather than the inability to unconsciously mimic facial expressions. Thus, we also tested participants’ abilities to recognize facial identity, and their lower-level visual and object processing capabilities.

## Methods

### Participants

Six individuals (four male) with a clinical diagnosis of Möbius sequence volunteered to take part in this study. A summary of each case is presented below, including estimated IQ according to the Wechsler Test of Adult Reading (WTAR [29]). Informed written consent was gathered prior to the experiment for all participants, and ethical approval was granted by the departmental Ethics Committee at Bournemouth University.

Participant MB1 is a 39 year-old male. He has an estimated IQ of 110 and was diagnosed with Möbius sequence when he was aged 6/12. BS has no lateral eye movement or convergence and no upward gaze, but can make some small downwards movements. He has worn glasses since four years of age. The only facial movement he can accomplish is some minimal puckering around the mouth. MB1 is also described in Calder et al. [24].

Participant MB2 is a 60 year-old male with an estimated IQ of 89. He was diagnosed with Möbius sequence at two years of age. He has had craniofacial reconstruction to improve his cheek bones and two face lifts, and also had a right trapezius flap for animation of the face, but without success. No eye movement was apparent upon examination, and there was minimal movement of the lower face near the mouth on the right side only.

MB3 is a 39 year-old female. Her estimated IQ is 124, and she was diagnosed with Möbius sequence shortly after birth. She has no facial movement at all, no abduction of the eyes, and limited downward movement.

MB4 was aged 49 years at the time of testing, and presented with an estimated IQ of 108. She was diagnosed with Möbius sequence at eight months of age, and has had repeated eye operations for squint, eye lid closure, strabismus, and blocked tear ducts. Upon examination there was no abduction of the eyes, but some slight adduction and elevation was possible. Looking down was incomplete but larger than other movements. Her facial movements were restricted to slight puckering round the mouth bilaterally.

MB5 is a 27 year-old male with an estimated IQ of 89. He was diagnosed with Möbius sequence aged 18/12. When he was a child he had little or no facial movement, but that has now considerably improved and his facial movements are large compared with the others in this study. He has reasonable eye shutting and can move the forehead, as well as make a smile and purse the lips. He has limited up and down gaze, but little adduction and no abduction.

MB6 was aged 43 years at the age of testing, and had only learnt of his diagnosis six years previously. He has an estimated IQ of 119. His eye movements show no abduction, limited convergence and elevation, but some depression. There is a small wrinkle around the right side of his mouth but no other facial movement.

Each Mobius participant’s performance was compared to that of one of three control groups. MB1, MB3, MB4 and MB6 were compared to an age-, gender- and IQ-matched control group containing four males and four females. Their average age was 48.5 years (SD = 4.8) and their average IQ was 117.0 (SD = 5.4). MB2 was compared to an age-, gender- and IQ-matched control group consisting of eight males with an average age of 56.3 years (SD = 8.3) and an average IQ of 92.8 (SD = 6.1). MB5 was also compared to an age-, gender- and IQ-matched control group that contained eight males with an average age of 21.4 years (SD = 3.49) and average IQ of 92.8 (SD = 3.5). Control participants were given a small monetary payment in exchange for their time.

### Design, Procedure and Statistical Analyses

All participants completed a series of neuropsychological tests assessing their expression, face, and object processing skills, in addition to their lower-level vision. They completed the tests in the same order within two separate testing sessions. We used Crawford and Howell’s [30] modified *t*-test for single case comparisons to assess whether each of the Mobius participant’s performance differed from the relevant control group on each test.

### Facial Expression Recognition

#### Ekman 60 Faces test

The Ekman 60 Faces test was presented to participants using the Facial Expressions of Emotion: Stimuli and Tests (FEEST) CD [31]. The test uses a range of photographs from the Ekman and Friesen [32] series of Pictures of Facial Affect to test recognition of basic facial expressions (i.e. those depicting anger, disgust, fear, happiness, sadness, and surprise). Participants are asked to complete six practice trials followed by 60 test trials. Stimuli are presented in a random order for five seconds per face, followed by a blank screen. Participants are required to use the mouse to click on-screen buttons representing each of the six basic emotions. The test is not timed – participants can take as long as they wish to make their response.

#### Emotional Hexagon test

The Emotional Hexagon test [31] assesses the recognition of more ambiguous facial expressions. Trials consist of morphed facial stimuli that were created from pairs of images depicting emotional expressions that are often confused (e.g. surprise and disgust). The original images were selected from the Ekman and Friesen [32] stimulus set, and were morphed to create 120 trials of varying difficulty (for more details on the image manipulation procedure see [33]). Participants are required to interpret the expressions in the same manner as described for the Ekman 60 Faces test.

#### Reading the Mind in the Eyes test

The ‘Mind in the Eyes’ test [34], [35] is a challenging test that assesses recognition of more complex expressions (e.g. correct responses include ‘panicked’, ‘playful’ and ‘upset’). In this test, 36 photographs of the eye region of the face are presented to participants. Four response options consisting of subtly different emotional states are presented alongside each image, and participants must decide which adjective best describes the emotional state of the model. This test was completed using paper-based print-outs of the test items, and participants were given an unlimited amount of time to provide their answers.

### Imagery

We assessed participants’ ability to imagine different emotional expressions by adopting the procedure used by Bowers et al. [36] and Jacobs et al. [28]. In this task, participants were required to imagine a face depicting a particular emotional expression (anger, fear, happiness or surprise), and to answer eight yes/no questions about the physical characteristics of each expression (e.g. ‘Are the lips curled up?’ ‘Are the nostrils dilated?’). An equal number of items had ‘yes’ and ‘no’ answers in each condition, and participants were instructed to answer the questions without making any facial movements (see Table S1 for all items presented in this test).

Participants were also given a control task to discern whether any imagery impairment may be general or expression-specific. Indeed, previous work has found that object and expression imagery are dissociable processes, such that impairment on one of the two tasks can be attributed to damage to the neural networks underpinning expression or object processing [28], [36]. Thus, we designed an object imagery questionnaire that was adapted from the methodology used by Eddy and Glass [37] and Bowers et al. [36]. In this test, participants were asked to respond to 20 questions that had yes/no or single-word answers about a particular object, e.g. ‘Is the date on a penny towards the top or the bottom?’ (see Table S2 for all items presented in this test).

### Facial Identity Processing

#### The Cambridge Face Perception Test (CFPT)

The CFPT [38] evaluates early stages of identity processing (i.e. those involved in face perception rather than face memory). Specifically, the test requires participants to sort a series of test faces in order of their similarity to a simultaneously-presented target face, thereby placing minimal demands on face memory. On each of eight upright trials, the six test faces are displayed from a different viewpoint than the target face, and have been morphed to contain different proportions of the target face: 28%, 40%, 52%, 64%, 76% and 88%. The participant has one minute to sort the faces according to their similarity to the target face. The deviation of the participant’s order from the correct order is calculated for each trial and summed to determine the total number of errors.

#### The Cambridge Face Memory Test (CFMT)

The CFMT is a test that is commonly used to assess memory for facial identity [39], [40], and to diagnose face recognition impairments in adults [41], [42], [43], [44], [45], [46], [47]. In the first part of the test, participants are introduced to six target faces and are then tested with 18 forced-choice items consisting of three faces. After a 20 second review of the target faces, participants are presented with 30 further triads of faces where they are required to select the target face, but images are taken from novel viewpoints or under novel lighting conditions. After a second opportunity to review the target faces, participants are presented with 24 additional test items with added noise (for full details see [48]). Importantly, in previous investigations the CFMT has demonstrated high reliability [40], [44] and both convergent and divergent validity [40], [44], [48].

#### Famous faces test

Memory for more robust facial representations was tested using a famous face test that has frequently been used in previous research examining face processing deficits in prosopagnosia [38], [42], [43]. In this test, participants view 60 faces of well-known celebrities, and are required to name or provide uniquely identifying biographical information about each face. Any faces that participants have low familiarity with from name cues (i.e. those they would not expect to recognize regardless of their face recognition ability) are removed from analysis and the proportion correct is adjusted accordingly. All faces have been cropped to remove the hair and any other external features which might cue recognition.

### Lower-Level Vision and Object Recognition

Lower-level vision was tested using four sub-tests from the Birmingham Object Recognition Battery (BORB) [49] that have been used in previous investigations of face processing ability [42], [43], [50], [51]. In the Length Match test, participants are required to judge whether two lines are of the same length; in the Size Match test they judge whether two circles are of the same size; in the Orientation Match test they decide whether two lines are parallel or not; and in the Position of the Gap Match test they decide whether the position of the gap in two circles is in the same position or not.

Basic object recognition was tested using the Object Decision test from the BORB. In this test, the participant is presented with a series of line drawings which depict animals or tools. In some trials, the drawings represent ‘unreal’ objects (i.e. the picture shows half of one object combined with half of another object) and the participant is asked to decide whether each of 128 drawings represents a real or unreal object. Memory for newly encoded objects was assessed using the Cambridge Car Memory Test (CCMT) [52]; a test that is identical in format to the CFMT (see above) but uses car rather than facial stimuli. While the design of the two tests is identical, the authors note that control means are not similar, indicating that performance is not directly comparable across the two tests.

## Results

### Facial Expression Recognition

In line with previous work [24], a varied pattern of performance was noted on the three expression recognition tests. All but one of the Möbius participants (MB4) achieved impaired scores on at least one of the three tests (see Table 1).

**Table 1:** Performance of Möbius participants on tests of emotional expression processing [31] in comparison to controls.

	Higher IQ					Older Low-IQ		Younger Low-IQ	
	Control Mean (SD)	MB1	MB3	MB4	MB6	Control Mean (SD)	MB2	Control Mean (SD)	MB5
*Ekman 60 faces:*									
Anger	8.63 (0.92)	9	0*	9	9	7.50 (1.9)	3	8.13 (1.55)	6
Disgust	7.75 (2.19)	5	10	7	6	7.38 (1.60)	10	8.13 (1.46)	4*
Fear	8.00 (1.31)	9	5	9	7	5.75 (2.92)	2	6.88 (1.96)	7
Happiness	10.00 (0.00)	10	10	10	9*	9.75 (0.71)	10	10.00 (0.00)	10
Sadness	8.50 (1.41)	4*	7	8	5*	7.88 (1.13)	9	8.75 (0.89)	9
Surprise	9.13 (0.83)	6*	7*	8	9	8.13 (1.64)	7	8.75 (1.04)	9
**Total**	**52.00 (4.90)**	**43**	**39** *	**51**	**45**	**46.38 (6.82)**	**41**	**50.63 (2.62)**	**45**
*Hexagon test:*									
Anger	18.63 (1.51)	19	13*	20	19	15.50 (6.02)	7*	18.00 (2.20)	2*
Disgust	17.00 (4.69)	13	20	18	18	13.13 (6.73)	13	18.50 (2.45)	7*
Fear	16.75 (3.45)	16	13	18	20	15.88 (3.80)	10	17.25 (2.12)	19
Happiness	19.88 (0.35)	16*	20	19	20	19.50 (0.93)	18	19.38 (1.19)	20
Sadness	19.75 (0.46)	14*	17*	20	19	18.50 (2.27)	17	19.13 (0.99)	20
Surprise	18.25 (2.05)	13*	14	15	14	17.13 (2.64)	18	18.13 (2.03)	20
**Total**	**110.25 (4.95)**	**91** *	**97** *	**110**	**110**	**99.63 (15.27)**	**83**	**110.38 (5.40)**	**88** *
*Mind in the Eyes:*	28.00 (2.51)	16*	20*	26	25	24.38 (4.75)	19	28.75 (3.54)	24

‘Higher IQ’ refers to Möbius participants and aged-matched controls with IQs within the higher range; ‘Older Low-IQ’ refers to the control group matched to MB2 according to age, IQ and gender; and ‘Younger Low-IQ’ refers to the control group matched to MB5 on the same measures.

* Represents impaired performance.

MB1 was impaired on all three tests. On the Ekman 60 Faces test he was impaired at the recognition of sadness and surprise, *t*(7) = 3.009, *p* = .020 and *t*(7) = 3.555, *p* = .009, although his overall score on this test was within the normal range. However, MB1's overall score on the Hexagon test was impaired *t*(7) = 3.666, *p* = .008, and he also demonstrated specific impairments in the recognition of happiness, sadness and surprise, *t*(7) = 10.452, *p* = .001, *t*(7) = 11.785, *p* = .001, and *t*(7) = 5.636, *p* = .001. MB1 also achieved a low score on the Mind in the Eyes test, *t*(7) = 4.507, *p* = .003.

MB2 did not demonstrate any impairments on the Ekman 60 Faces and Mind in the Eyes tests, but did achieve a low score in the recognition of anger in the Hexagon test, *t*(7) = 6.600, *p* = .001.

MB3 showed impairments in all three tests. Specifically, she achieved a low overall score on the Ekman 60 Faces test, *t*(7) = 2.501, *p* = .041; with specific impairments in the recognition of anger and surprise, *t*(7) = 8.844, *p* = .001 and *t*(7) = 2.419, *p* = .046. Further, MB3 also achieved a low overall score on the Hexagon test, *t*(7) = 2.524, *p* = .040, with specific impairments in the recognition of anger and sadness, *t*(7) = 3.515, *p = *.010 and *t*(7) = 5.636, *p* = .001. Her score also fell into the impaired range on the Mind in the Eyes test, *t*(7) = 3.005, *p* = .020.

MB5 demonstrated impairments on the Ekman 60 Faces and Hexagon tests, but achieved a normal score on the Mind in the Eyes test. Specifically, he was impaired at the recognition of disgust in the Ekman 60 faces test, *t*(7) = 2.667, *p* = .032; although his overall score was within the normal range. He also achieved a low overall score in the Hexagon test, *t*(7) = 3.907, *p = *.006; with specific impairments in the recognition of anger and disgust, *t*(7) = 6.857, *p = *.001 and *t*(7) = 4.425, *p = *.003.

MB6 struggled to recognize happiness and sadness in the Ekman 60 Faces test, *t*(7) = 9.428, *p* = .001 and *t*(7) = 2.340, *p* = .051; although his overall score was within the normal range. He also achieved normal scores on the Hexagon and Mind in the Eyes tests.

### Imagery

MB1, MB4, MB5 and MB6 achieved normal scores on both the expression and object imagery tasks (see Table 2). Only one participant (MB2) was found to be impaired on the expression imagery test, *t*(7) = 2.847, *p* = .012, and he showed a corresponding impairment on the object imagery test, *t*(7) = 3.130, *p* = .017, indicating he has a more general imagery impairment. Interestingly, MB3 was impaired at the object imagery task, *t*(7) = 3.720, *p* = .007, but did not show an impairment on the expression imagery task.

**Table 2:** Performance of Möbius participants on the imagery tasks in comparison to matched control groups.

	Normal IQ					Older Low-IQ		Younger Low-IQ	
	Controls	MB1	MB3	MB4	MB6	Controls	MB2	Controls	MB5
Expression imagery	24.25 (2.60)	18	23	21	25	22.13 (2.03)	16*	24.25 (2.82)	22
Object imagery	19.13 (1.30)	16	14*	17	18	18.25 (1.28)	14*	18.13 (0.83)	18

‘Higher IQ’ refers to Möbius participants and aged-matched controls with IQs within the higher range; ‘Older Low-IQ’ refers to the control group matched to MB2 according to age, IQ and gender; and ‘Younger Low-IQ’ refers to the control group matched to MB5 on the same measures.

* Represents impaired performance.

### Facial Identity Processing

**Figure 1:**
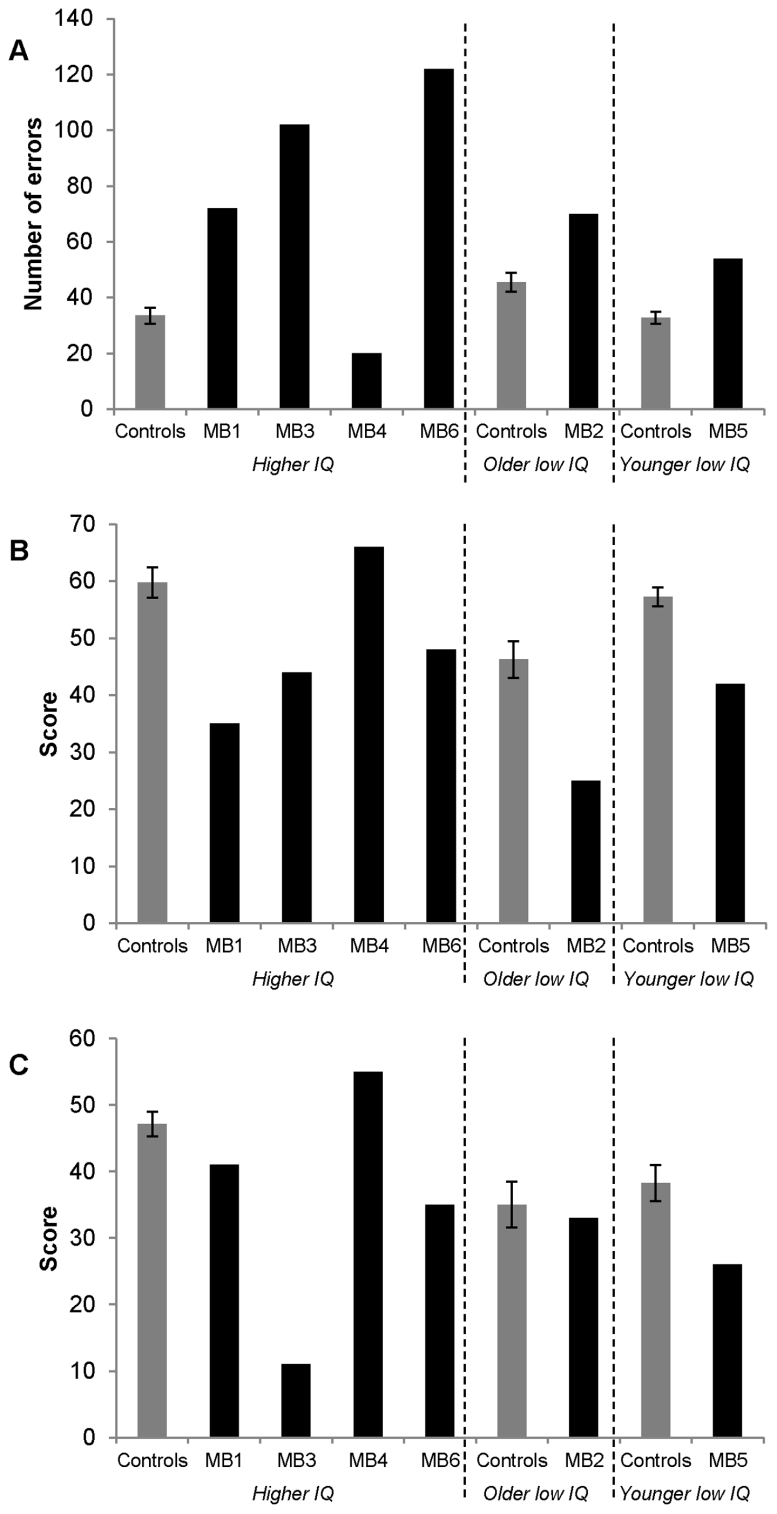
Performance on (A) the CFPT, (B) the CFMT and (C) a famous faces test. ‘CFPT’ refers to the Cambridge Face Perception Test [38] and ‘CFMT’ to the Cambridge Face Memory Test [48]. ‘Higher IQ’ refers to Möbius participants and aged-matched controls with IQs within the higher range; ‘Older Low-IQ’ refers to the control group matched to MB2 according to age, IQ and gender; and ‘Younger Low-IQ’ refers to the control group matched to MB5 on the same measures.

Two participants (MB2 and MB4) performed within the normal range on all tests. However, the other four participants were impaired on at least one of the three facial identity tests (see Figure 1). Indeed, MB1, MB3, MB5 and MB6 were all impaired at face perception in the CFPT, *t*(7) = 4.358, *p* = .003, *t*(7) = 7.753, *p = *.001, *t*(7) = 3.317, *p* = .013, and *t*(7) = 10.017, *p* = .001, respectively. Further, MB1 and MB5 achieved impaired scores on the CFMT, *t*(7) = 3.136, *p* = .016 and *t*(7) = 3.112, *p* = .017. In addition, MB2, MB3 and MB6 achieved low scores on this test that were at least 1.5 standard deviations below the control mean (see Figure 1). One participant (MB3) achieved an impaired score on the famous face test, *t*(7) = 6.355, *p* = .001.

### Lower-Level Vision and Object Recognition

Three Möbius participants (MB4, MB5 and MB6) achieved normal scores on the four tests of lower-level vision from the BORB (see Table 3). However, MB1 achieved a low score on the Length Match test, *t*(7) = 3.241, *p* = .014; MB2 achieved a low score on the Orientation Match test, *t*(7) = 6.169, *p* = .001; and MB3 scored within the impaired range on the Size Match test, *t*(7) = 3.406, *p* = .011. Three of the Möbius participants were impaired on the object processing tests (see Table 3). Specifically, MB2 and MB4 performed poorly on the Object Decision test, *t*(7) = 6.760, *p* = .001 and *t*(7) = 3.209, *p* = .015; and MB1 achieved a low score on the CCMT, *t*(7) = 2.401, *p* = .047.

**Table 3:** Performance of Möbius participants on lower-level vision and object processing tests in comparison to controls.

	Normal IQ					Older Low-IQ		Younger Low-IQ	
	Controls	MB1	MB3	MB4	MB6	Controls	MB2	Controls	MB5
*BORB:*									
Length match	27.50 (1.60)	22*	26	25	26	26.38 (1.20)	27	26.63 (1.06)	25
Size match	26.75 (1.04)	26	23*	26	27	27.00 (0.76)	26	26.13 (2.17)	25
Orientation match	29.38 (4.69)	20	27	25	30	26.75 (1.49)	17*	26.75 (0.89)	25
Gap match	33.63 (5.10)	34	37	37	37	32.88 (3.44)	38	34.00 (3.42)	37
Object decision	119.30 (3.32)	114	112	108*	124	116.30 (3.11)	94*	116.80 (4.53)	111
*CCMT*	59.88 (8.20)	39*	40	42	70	58.50 (9.59)	36	63.25 (9.68)	46

‘Higher IQ’ refers to Möbius participants and aged-matched controls with IQs within the higher range; ‘Older Low-IQ’ refers to the control group matched to MB2 according to age, IQ and gender; and ‘Younger Low-IQ’ refers to the control group matched to MB5 on the same measures. ‘BORB’ refers to the Birmingham Object Recognition Battery [49]; and ‘CCMT’ to the Cambridge Car Memory Test [52].

* Represents impaired performance.

## Discussion

This investigation tested embodied simulation theories of emotional expression processing by examining the recognition and imagery of facial expressions in six individuals with Möbius sequence. Impairments in the recognition of facial expressions were noted in five participants, although these deficits were not absolute and mostly considered of below-control-level performance rather than a complete inability to perform the tasks. However, only one participant was impaired in the expression imagery test, and that individual also performed poorly on a corresponding object imagery test, suggesting he had a more generalized imagery impairment. Further, five participants were impaired on at least some measures of facial identity, object or lower-level visual processing.

First, it is notable that five of the six Mobius participants were impaired on at least some measures of expression recognition, and this pattern of findings fit well with those of Calder et al. [24]. Indeed, these authors used two of the three expression recognition tests used in the current study, and also reported mild impairments in their Möbius participants. However, Bogart and Matsumoto [23] did not find any evidence of expression recognition impairments in 37 individuals with Möbius sequence, whereas Giannini et al. [22] reported a single participant who was completely unable to perform an expression recognition task. It is likely that the different pattern of findings across studies results from the different methodologies used to assess expression recognition. However, it is important to note that the tests used by us and by Calder et al. have been used successfully in numerous investigations to detect both mild and severe expression recognition difficulties in patients with a range of aetiologies [33], [53]. Thus, we agree with the conclusion of Calder et al. [24] that expression recognition difficulties are prevalent in Möbius sequence but are not absolute, providing evidence against the strongest form of the embodied simulation theory of expression recognition. Indeed, these theories suggest that individuals with Möbius sequence should not be able to recognize facial expressions at all, yet there clearly is at least some residual ability to recognize expressions in all six of the participants reported here.

Second, it is also of theoretical interest that only one of the Möbius participants was impaired on the expression imagery test (and importantly the impaired participant appeared to have a more generalized imagery deficit). This finding can be interpreted as further evidence against the reverse simulation theories of expression processing, and specifically the hypothesis that feedback from the face is also necessary for the imagery of facial expressions of emotion [25], [26], [27]. However, these theories also assume that expression perception and expression imagery are linked, yet, the data presented here does not directly support this hypothesis. While it could be argued that the imagery test we used was not sensitive enough to detect mild impairments, this is unlikely as the same test has been used in previous work to detect expression imagery impairments in participants with Parkinson’s disease [28]. It should be acknowledged, however, that our expression recognition and expression imagery tests were not explicitly matched for difficulty.

Alternatively, it may be that case that feedback from facial movement is less important for expression imagery than perception, although previous work has reported a strong correlation between the three measures [28]. One might then ask whether alternative co-opted theories of emotion processing are more successful in accounting for the pattern of findings reported here. For instance, it has been posited that the same neural regions are activated when a person feels a particular emotion as when they observe another person experiencing that emotion [54], [55], [56]. Evidence supporting this hypothesis comes from patient studies reporting impairment in the recognition of emotional expressions following lesions affecting the insulae and nearby structures [55], [56]. Further, Wicker et al. [54] reported similar levels of activation in the insula when participants viewed facial expressions of disgust compared to when they inhaled odorants that produced strong feelings of disgust. Thus, rather than suggesting expression recognition occurs following the initiation of a facial motor representation and its associated somatosensory consequences, this theory suggests that the observer actually shares the emotion with the observed individual via the activation of brain regions underpinning the experience of that emotion. Many aspects of mirror neuron theory are still being debated, and it is unclear whether the same mirror neurons are activated when a person imagines an expression as when they experience or perceive that expression. If it is assumed that expression imagery does not activate relevant mirror neurons, the pattern of findings reported here (i.e. impaired expression recognition but preserved expression imagery) can be accommodated. However, there is some evidence to suggest that the same mirror neurons are involved in imagery as in perception or experience of an action [57], and if this is the case, it is difficult to reconcile the theory with the current findings. Clearly, further neuroimaging work is needed to resolve this issue.

An alternative explanation is that the impairments in expression recognition may largely result from more generalized perceptual abnormalities in Möbius syndrome. Indeed, four of the participants also displayed deficits in the recognition of facial identity, and four were also impaired in other tests examining object processing and lower-level vision. Hence, it may simply be that mild impairments in lower-level vision are underpinning the impairments noted on tests of expression recognition (as discussed above, these impairments were not absolute), but would not interfere with tests of visual imagery. These perceptual difficulties may simply relate to the absence of eye movements in the condition, as suggested by Calder et al. [24]. Indeed, work using eye movement technology suggests this process is particularly important in face processing [42], [43], [58], [59], and it is of note that deficits in visual perception have been used as an explanation for face recognition deficits in other developmental conditions. For instance, evidence from people who were born with congenital infantile cataracts that were removed at an early age suggests that early visual experience with faces is critical for the development of normal face processing skills [60]. We can presume that such an explanation can account for the face processing impairments noted in five of the six Möbius participants in the current study, as the lack of eye movements and poor vision in all participants is likely to have prevented them from having normal visual experience with faces, particularly in their early years.

Such an explanation may also account for two further observations of our data. First, it is of interest that only some expressions were impaired in each participant, and additionally that the same expressions were not affected across participants. This pattern may be explained by the hypothesis that individuals with Möbius have generalized perceptual difficulties rather than damage to specific emotion systems. Second, the Möbius participants also performed better in the famous faces task as opposed to the CFMT and CFPT. While there were time restrictions in sections of both the latter tests, no such restrictions were imposed in the famous face test, where participants were allowed as much time as necessary to provide their answer. Likewise, the Mind in the Eyes test does not involve a timed component, whereas the Ekman 60 Faces and Emotional Hexagon tests both have time restrictions in the presentation of the faces. Interestingly, only one participant was impaired on the famous faces test, and only two on the Eyes in the Mind test. Hence, it may be that slowed perceptual processing is contributing to at least some of the impairments noted here.

Of course, it may be that there are independent causes of the facial identity and expression recognition impairments, and this issue can be further informed by neuroimaging studies examining the key structures implicated in these processes. Indeed, it may be that the affective system is disrupted in Möbius sequence (regardless of the proposed involvement of mirror neurons), and this is bringing about additional problems in affect recognition that are over and above those caused by impairments in general visual processing. In any case, a novel finding reported here is that individuals with Möbius sequence may also have impairments in recognizing facial identity, and this has important practical implications for management of the condition.

Finally the performance of participant MB4 warrants discussion. MB4 was the only participant who demonstrated normal performance on all tests, and her case suggests that face processing impairments are not always present in Möbius sequence despite perhaps being indicative of the condition. After we completed testing and had analyzed our data, we discussed our findings with MB4. She reported that she has always been extremely interested in faces, and enjoys looking at them. Indeed, we noted that MB4 spent an unusually long period of time examining our own faces during the testing session. It is possible that MB4’s interest in faces may be one factor that has helped her to overcome any face processing difficulties.

In conclusion, the findings reported here provide evidence against embodied simulation theories of emotional expression processing. However, it is important to note that both facial expression and facial identity processing deficits appear to be characteristic of many (but not all) people with Möbius sequence. While these may be mild in some individuals, the findings of this investigation suggest that more profound identity recognition impairments may be more common in the disorder than previously envisaged and should be considered by those caring for people with the condition.

## Supporting Information

Table S1 **Items in the expression imagery test.**
(DOCX)

Table S2 **Items in the object imagery test.**
(DOCX)
