# Supplementary material for: First Report of Generalized Face Processing Difficulties in Möbius Sequence
Source: PLoS One. 2013 Apr 24;8(4):e62656. doi: 10.1371/journal.pone.0062656 (PMC3634771; doi:10.1371/journal.pone.0062656)
Supplement: Table S1 — Items in the expression imagery test. (DOCX) [file pone.0062656.s001.docx]

*Table S1:* Items in the expression imagery test.

| **Item** | **Expression** | **Question** | **Answer** |
| --- | --- | --- | --- |
| 1 | Sadness | Are the inner corners of the eyebrows raised? | No |
| 2 |  | Are the cheeks raised? | No |
| 3 |  | Is the brow furrowed? | Yes |
| 4 |  | Do the eyelids droop? | Yes |
| 5 |  | Is the jaw dropped? | No |
| 6 |  | Are the nostrils dilated? | No |
| 7 |  | Is the nose relaxed? | Yes |
| 8 |  | Are the eyes open wide? | No |
| 9 | Fear | Are the eyebrows raised? | Yes |
| 10 |  | Are the cheeks raised? | No |
| 11 |  | Do the eyelids droop? | No |
| 12 |  | Are the eyes open wide? | Yes |
| 13 |  | Is the jaw clenched? | No |
| 14 |  | Are the nostrils dilated? | Yes |
| 15 |  | Is the skin around the eyes wrinkled? | No |
| 16 |  | Is the mouth open? | Yes |
| 17 | Happiness | Is the skin around the eyes wrinkled? | Yes |
| 18 |  | Are the inner corners of the eyebrows raised? | No |
| 19 |  | Are the cheeks raised? | Yes |
| 20 |  | Is the brow relaxed? | Yes |
| 21 |  | Is the jaw lowered? | No |
| 22 |  | Are the lips stretched? | Yes |
| 23 |  | Are the nostrils dilated | No |
| 24 |  | Are the eyes open wide? | No |
| 25 | Anger | Are the inner corners of the eyebrows raised? | No |
| 26 |  | Are the cheeks raised? | No |
| 27 |  | Is the brow furrowed? | Yes |
| 28 |  | Are the eyes narrowed? | Yes |
| 29 |  | Is the jaw clenched? | Yes |
| 30 |  | Are the nostrils dilated? | Yes |
| 31 |  | Is the skin around the eyes wrinkled? | No |
| 32 |  | Are the lips stretched? | No |
