# Supplementary material for: First Report of Generalized Face Processing Difficulties in Möbius Sequence
Source: PLoS One. 2013 Apr 24;8(4):e62656. doi: 10.1371/journal.pone.0062656 (PMC3634771; doi:10.1371/journal.pone.0062656)
Supplement: Table S2 — Items in the object imagery test. (DOCX) [file pone.0062656.s002.docx]

*Table S2:* Items in the object imagery test.

| **Item** | **Question** | **Answer** |
| --- | --- | --- |
| 1 | What is higher off the ground, a horse’s knee or the top of its tail? | Tail |
| 2 | Is the date on a penny towards the top or bottom? | Bottom |
| 3 | Which number between 1 and 9 looks the most similar to the letter ‘S’? | 5 |
| 4 | Is a standard pin used in sewing longer than 5cm in length? | No |
| 5 | Are the two large wheels on a tractor at the front or the back? | Back |
| 6 | Is the hot water tap on a sink normally on the left or the right? | Left |
| 7 | Is a grapefruit larger than an orange? | Yes |
| 8 | Is the symbol for ‘degrees’ a small circle or an apostrophe? | Small circle |
| 9 | Is the colour brown or yellow darker than orange? | Brown |
| 10 | A right handed batsman places his left or right side towards the bowler? | Left |
| 11 | To open a jar do you turn the lid clockwise or anti-clockwise? | Anti-clockwise |
| 12 | When you look at a world map is Russia to the east or west of Germany? | East |
| 13 | On a standard QWERTY keyboard is the key to type a question mark towards the top or bottom? | Bottom |
| 14 | Is a cat’s tail longer than its legs? | Yes |
| 15 | What is larger, a strawberry or a raspberry? | Strawberry |
| 16 | When you set the table, is the fork placed to the right or left? | Left |
| 17 | On the left foot is the big toe on the left or right? | Right |
| 18 | On a compass are there 180 or 270 degrees between North and West? | 270 |
| 19 | Which is smaller, a thimble or a cotton reel? | Thimble |
| 20 | When posting a letter is the stamp placed on the top right or top left of the envelope? | Right |
